# Influence of intergenerational social mobility on brain structure and global cognition: findings from the Whitehall II study across 20 years

**DOI:** 10.1093/ageing/afae221

**Published:** 2024-10-12

**Authors:** Yingxu Liu, Benjamin Thyreau, Yuehua Cui, Ye Zhang, Yasuko Tatewaki, Yasuyuki Taki

**Affiliations:** Department of Aging Research and Geriatric Medicine, Institute of Development, Aging and Cancer, Tohoku University, Sendai, Japan; Smart-Aging Research Center, Institute of Development, Aging and Cancer, Tohoku University, Sendai, Japan; Department of Statistics & Probability, Michigan State University, East Lansing, MI, USA; Department of Aging Research and Geriatric Medicine, Institute of Development, Aging and Cancer, Tohoku University, Sendai, Japan; Department of Aging Research and Geriatric Medicine, Institute of Development, Aging and Cancer, Tohoku University, Sendai, Japan; Department of Geriatric Medicine and Neuroimaging, Tohoku University Hospital, Sendai, Japan; Department of Aging Research and Geriatric Medicine, Institute of Development, Aging and Cancer, Tohoku University, Sendai, Japan; Smart-Aging Research Center, Institute of Development, Aging and Cancer, Tohoku University, Sendai, Japan; Department of Geriatric Medicine and Neuroimaging, Tohoku University Hospital, Sendai, Japan

**Keywords:** intergenerational social mobility, brain grey matter volume, cortical thickness, cognitive ageing, older people

## Abstract

**Background:**

Whether changes in socioeconomic position (SEP) across generations, i.e. intergenerational social mobility, influence brain degeneration and cognition in later life is unclear.

**Objective:**

To examine the association of social mobility, brain grey matter structure and global cognition.

**Methods:**

We analysed T1 brain MRI data of 771 old adults (69.8 ± 5.2 years) from the Whitehall II MRI substudy, with MRI data collected between 2012 and 2016. Social mobility was defined by SEP changes from their fathers’ generation to mid-life status. Brain structural outcomes include grey matter (GM) volume and cortical thickness (CT) covering whole brain. Global cognition was measured by the Mini Mental State Examination. We firstly conducted analysis of covariance to identify regional difference of GM volume and cortical thickness across stable high/low and upward/downward mobility groups, followed with diagonal reference models studying the relationship between mobility and brain cognitive outcomes, apart from SEP origin and destination. We additionally conducted linear mixed models to check mobility interaction over time, where global cognition was derived from three phases across 2002 to 2017.

**Results:**

Social mobility related to 48 out of the 136 GM volume regions and 4 out of the 68 CT regions. Declined volume was particularly seen in response to downward mobility, whereas no independent association of mobility with global cognition was observed.

**Conclusion:**

Despite no strong evidence supporting direct influence of mobility on global cognition in later life, imaging findings warranted a severe level of neurodegeneration due to downward mobility from their father’s generation.

## Key Points

- We examined how ‘fall from grace’ or ‘rise from rags’ from the previous generation influences personal brain cognition.
- ‘Falling from grace’ projects particular burden on brain neurodegeneration, with declined grey matter volume and thickness.
- In contrast, mobility does not independently influence global cognition and its changes over time.

## Introduction

Time is equal, but ageing is not; individuals face different cognitive risks while ageing. Recent analysis shows that older adults in lower socioeconomic positions (SEP) have up to a 60% higher dementia rate >12 years [1]. Low SEP’s deleterious effects begin from the brain development phase (refer to review [2]); it determines the regulation of the hypothalamic–pituitary–adrenal (HPA) axis with the release of glucocorticoids. The prolonged dysregulation of the HPA axis and high glucocorticoid level draw back the functional and structural development of the brain, particularly in the regions responsible for memory (the hippocampus; see [3, 4, 5]), executive function (superior frontal gyrus of the prefrontal cortex; see [6, 7, 8]) and social emotion (the insula and amygdala; see [9, 10]). Unfortunately, such a brain in ‘rags’ is likely to persist: worse cognitive performance and memory are not only seen during childhood but also evident in later life [11, 12, 13, 14].

However, this is not to say that such cognitive brain vulnerability cannot be compensated. Participants may benefit from ‘rising from the rags’, upwardly mobile from the original generation. In hypothesis, climbing the social ladder provides individuals with a cognitively stimulating life environment and therefore enriches one’s capability (i.e. knowledge of a healthy lifestyle and access to health professionals) to cope with the cognitive impediment brought by earlier life [15]. Some observational studies have confirmed the protective role of upward mobility against cognitive decline [16, 17, 18, 19], but neuroimaging evidence explains that such association is scarce. Only two MRI studies so far linked social mobility to brain structure in later life, and found downward mobility related to grey matter volume reduction and thinning in Alzheimer’s disease–sensitive areas including the hippocampus and prefrontal cortex [20, 21]. From these findings, we could assume variant risks of cognitive decline and brain neurodegeneration due to mobility in SEP across generations. However, the previous imaging analysis that focuses on a predefined region of interest may omit important brain regions relevant to early cognitive disturbances. Thus, this study aims to fill this gap using Whitehall II imaging substudy data, focusing on the whole brain to identify vulnerable regions to social mobility and verify association of mobility on cognition, to distinguish adverse and/or protective roles in mobile life experiences.

## Method

### Sample overview

We derived data from the Whitehall II imaging substudy (2012–16, ClinicalTrials.gov, NCT03335696; UKRI: G1001354) [22]. The Whitehall II prospective cohort study was originally conducted from 1985 to 1988 among 10 308 British civil servants aged 35–55 years [23]. Sociodemographic, behavioural and health-related factors were assessed with questionnaires and clinical examinations approximately every 4 to 5 years (ClinicalTrials.gov: NCT00005680: UKRI: MR/R024227/1).

A total of 800 participants were randomly selected to participate in the imaging substudy, in which structural MRI scans were performed at the University of Oxford between 2012 and 2016. Of these, 771 cognitively intact participants with validated intensity normalised T1 structural MRI scans were included in our study, with a mean age of 69.8 ± 5.2 years. Participants were predominantly male (81%).

### Intergenerational social mobility classification

Both father’s class (collected at the baseline survey, participants’ age range: 35–55 years) and individual’s mid-life (45–68 years) SEPs were measured by the Registrar General’s Social Classes scheme, which has six levels ranging from the lowest to the highest cognitive demands, from unskilled workers (i.e. labourers, cleaners) to professional occupations (i.e. doctors, lawyers). We provided participants’ SEP distribution and detailed definitions in Supplemental Table 1. Due to the small cell size in lower SEP levels and the absence of the lowest level six, we separated high and low SEP by the third level (skilled non-manual occupations, i.e. clerks and cashiers). Upward social mobility was defined as the father belonging to the lower three levels and the individual belonging to the higher three levels; downward social mobility was the opposite. Stable high SEP was defined as both father and individual belonging to the higher three levels, whereas stable low SEP was the opposite. This definition follows the previous Whitehall framework, and also demonstrated sensitivity in predicting cardiovascular disease morbidity, obesity, type 2 diabetes and inflammatory markers [24, 25, 26].

### Brain structural outcomes

Normal brain ageing involves GM volume loss and cortical thinning, but each is influenced by different genetic factors [27]. As such, we analysed GM volume and cortical thickness (CT) separately. To refine the locations, we parcelled 136 regions on GM volume by the Neuromorphometrics Atlas (http://www.neuromorphometrics.com) and 68 regions on CT by the Desikan–Killiany Atlas [28] from T1 structural MRI scans. MRI acquisition and preprocessing are provided in the supplementary text. The global brain structure outcomes are calculated as total GM volumes and mean CT across all brain regions.

### Cognitive outcomes

Global cognition was evaluated using the 30-point Mini Mental State Examination (MMSE) in Phase 12 (2015–17) [29]. The other three times prior to the latest evaluation were measured from individual mid-life in Phase 7 (2002–04), Phase 9 (2007–09), to Phase 11 (2012–13).

### Analytic strategy

Firstly, we conducted analysis of covariance (ANCOVA) tests to explore regions with structural variations in GM volume and CT in different mobility groups. We corrected for scanner type, participant age at imaging substudy, gender, education years and total intracranial volume. The Benjamini–Hochberg procedure was adopted to account for multiple comparison problems, with the false discovery rate (FDR) correction threshold set to 0.05 [30]. Regions passing this threshold were inputted in further pair-wise Tukey tests across mobility groups.

In the second step, we applied Sobel’s diagonal reference models (DRMs) to verify the association of mobility on global brain structure outcomes and global cognition, beyond the SEP origin and destination [31]. We used the ‘drm’ package developed by Kaiser [32] in the Stata environment with non-linear least square estimations. We constrained the weight parameters for SEP origin (father) and destination (self) between 0 and 1, ensuring that their sum equalled 1. Dummy variables indicating upward or downward mobility were created and added to the DRMs, with adjustments for age and gender. Additionally, we created a mobility distance by contrasting the SEP level from the father. We also estimated ‘any mobility’ (a dummy variable indicating either mobilisation or stability); however, due to high collinearity with other mobility variables, the DRM constructions encountered non-concave issues. Thus, we included it only in the sensitivity analysis, with adjusting other socio-lifestyle covariates (detailed in the supplemental text) including marital status (cohabiting/married or not), education mobility (differences in the age of finishing education from the father), fluid intelligence, and lifestyle disorders such as depression, diabetes, hypertension and depression.

Finally, we estimated the influence of social mobility (ref. stable high SEP) on global cognition over time in traditional methods using linear mixed models. Restricted maximum-likelihood estimation with unconstructed covariance was adopted, accounting for partially missing and unbalanced data [33]. Due to ethical restrictions, the exact age of participants in each study phase is not available [34]. Therefore, we used the mean years since the baseline survey (1985) as the time proxy [35]. Age, gender, and global GM volume and CT were adjusted.

All analyses were performed using Stata 17 (StataCorp) on the DPUK desktop. The alpha level was set as 5% in two-sided tests.

### Standard protocol approvals, registrations and patient consents

Research ethics approval of the Whitehall Study was obtained from the University College London ethics committee. At each wave of data collection, participants provided written informed consent. The Whitehall imaging substudy was approved by the Oxford Medical Science Division Interdisciplinary Research Ethics Committee (MSD-IDREC-C1-2011-71). Data access and analysis were approved by the Dementias Platform UK (DPUK) [34] at the time of the study.

## Results

### Sample characteristics

A total of 144 participants were classified in the stable high SEP group, 535 in the upward mobility group, 17 in the stable low group and 75 in the downward mobility group (See Table 1). There were no significant differences in age or gender across the groups, nor in total intracranial volume. Compared to stable high SEP, upward mobility participants had marginally less education. Those in the stable low SEP group reported more obesity, and those in downward mobility reported more depression cases.

**Table 1:** Sample characteristics (*N* = 771), per social mobility group^a^

	Stable high (*N* = 144)	Upward mobility (*N* = 535)	Stable low (*N* = 17)	Downward mobility (*N* = 75)
Age at MRI, (SD)	69.6 (5.1)	70.0 (5.2)	68.1 (5.0)	69.0 (5.0)
Male, %	119 (82.6)	431 (82.6)	15 (88.2)	57 (76.0)
Married or cohabiting, %	109 (75.7)	418 (78.3)	16 (94.1)	60 (80.0)
Year of education	17.0 (3.4)	**16.5 (4.7)** **^†^**	16.8 (3.5)	17.5 (4.0)
Total intracranial volume, cm^3^	1552.1 (143.0)	1537.6 (135.4)	1541.9 (184.8)	1520.0 (136.8)
Mini Mental State Examination^a^	28.6 (1.7)	28.5 (1.5)	28.8 (1.0)	28.4 (1.4)
Obesity, %	20 (13.8)	94 (17.6)	**5 (29.4)** **^†^**	**7 (9.3)** ^*^
Depression, %	8 (5.6)	40 (7.5)	0 (0)	**12 (16.0)** ^*^
Hypertension, %	78 (54.2)	216 (53.5)	12 (70.6)	35 (47.9)
Diabetes, %	9 (6.3)	52 (9.8)	2 (11.8)	6 (8.0)

a Mini Mental State Examination Score at first valuation

b Data are presented as mean (standard deviation, SD) unless otherwise indicated. χ^2^ tests were conducted for variables of proportion and *t*-tests for continuous variables compared with stable high socioeconomic position group. Bold variable suggests statistical significance at ^†^*P* < .1, ^*^P < .05.

### Brain structural variations by mobility patterns

**Figure 1:**
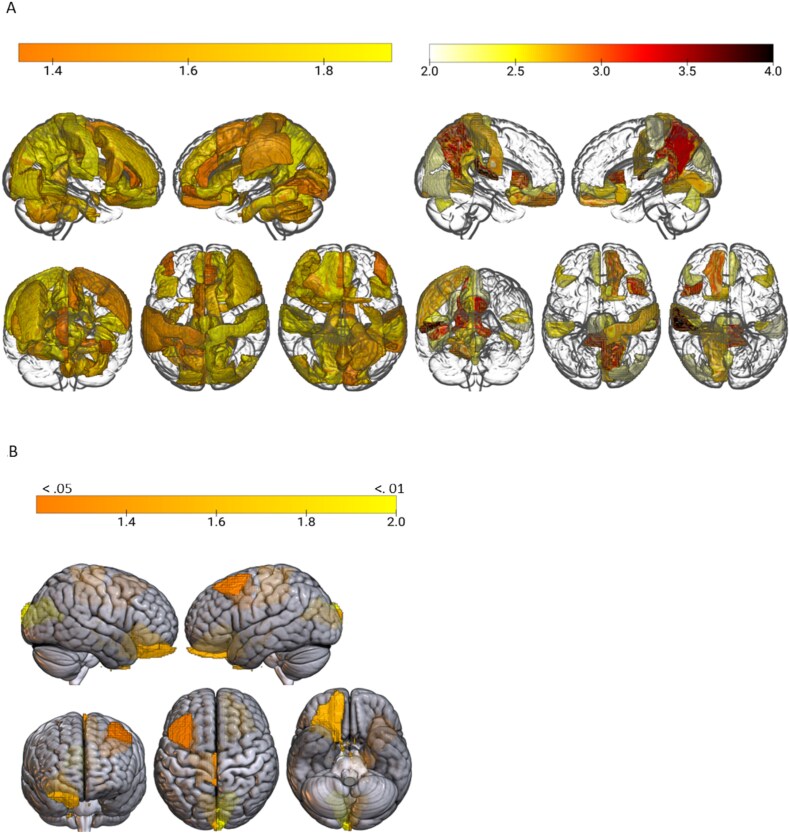
(A) Regions of grey matter volume related to social mobility. Regional density indicated FDR-corrected *P*-value from ANCOVA <0.05 level (log-transformed, adjusted for scanner, age, gender, education and total intracranial volume). (B) Regions of grey matter cortical thickness related to social mobility. Colour density indicated FDR-corrected *P*-value from ANCOVA <0.05 level (log-transformed, adjusted for scanner, age, gender, education and total intracranial volume).

The ANCOVA revealed significant differences in 48 out of 136 regions of GM volume and 4 out of 48 cortical thickness regions across mobility groups, after FDR correction. Figure 1 shows the projections of ANCOVA results at the whole brain level. Table 2 presents the pair-wise differences in the detected regions, along with the standard error of the Tukey post hoc analyses. For ease of reading, we have highlighted the top 5 regions of GM volume.

**Table 2:** Difference of regional grey matter volume and cortical thickness in social mobility groups (*N* = 771)^a^

	Stable high vs. downward mobility	Stable low vs. downward mobility	Upward vs. downward mobility	Stable low vs. high	Upward mobility vs. stable high	Upward mobility vs. stable low
Grey matter volume, cm^3^ (SE) top 5 regions						
Left superior frontal gyrus	.72 (.20)^**^^*^	.62 (.37)	.47 (.17)^*^	−.10 (.35)	−.25 (.13)	−.16 (.34)
Left posterior insula	.09 (.03)^**^	.12 (.05)	.06 (.02)	.03 (.05)	−.03 (.02)	−.06 (.05)
Left fusiform gyrus	.27 (.09)^**^	.30 (.18)	.21 (.08)^*^	.03 (.17)	−.06 (.06)	−.08 (.16)
Right posterior insula	.10 (.03)^**^	.07 (.06)	.07 (.03)^*^	−.03 (.05)	−.03 (.02)	.005 (.05)
Left precentral gyrus medial segment	.14 (.04)^**^	.004 (.08)	.07 (.04)	−.14 (.07)	−.07 (.03)	.07 (.07)
Sum of 48 regions related to social mobility	8.45 (2.73)^**^	5.02 (5.14)	5.83 (2.36)^*^	−3.43 (4.91)	−2.61 (1.80)	.82 (4.72)
Cortical thickness, mm (SE)						
Right cuneus	.045 (.021)	−.092 (.039)	.026 (.018)	−.136 (.037)^**^^*^	−.018 (.136)	.117 (.035)^**^
Right lateral orbitofrontal	.056 (.018)^**^	−.008 (.033)	.039 (.015)^*^	−.064 (.031)	−.016 (.012)	.048 (.031)
Left cuneus	.017 (.021)	−.091 (.040)	−.010 (.018)^*^	−.109 (.038)^*^	−.027 (.014)	.082 (.037)
Left paracentral lobule	.084 (.031)^*^	−.013 (.058)	.044 (.027)	−.096 (.056)	−.039 (.020)	.057 (.053)
Sum of 4 regions related to social mobility	.20 (.07)^*^	−.20 (.13)	.10 (.06)	−.41 (.13)^**^	−.10 (.05)	.30 (.12)

a Adjusted *P*-values using Tukey pair-wise results

^*^ *P* < .05, ^**^P < .01, ^***^P < .001

Structural variations were mostly seen in GM volume regions, notably the left superior frontal gyrus (*F* = 8.68, *P*_fdr_ < .001), left posterior insula (*F* = 6.58, *P*_fdr_ < .001; right: *F* = 6.36, *P*_fdr_ < .001), left fusiform gyrus (*F* = 6.45, *P*_fdr_ < .001) and left precentral gyrus medial segment (*F* = 5.59, *P*_fdr_ = .002). The sum value of these regions varied across groups, with downward mobility showing the least (209.44 cm^3^) compared to stable high (217.88 cm^3^, diff.: 8.45 cm^3^, *P* < .01) and upward mobility (215.27 cm^3^, diff.: 5.83 cm^3^, *P* < .05).

For cortical thickness, significant differences were observed in the right and left cuneus (*F* = 5.44, *P*_fdr_ < .001; *F* = 3.31, *P*_fdr_ < .05), right lateral orbitofrontal region (*F* = 4.14, *P*_fdr_ < .05) and left paracentral lobule (*F* = 3.05, *P*_fdr_ < .05), with overall smaller effect sizes. Total CT values also differed, with stable low SEP having the lowest (8.14 mm) and stable high SEP the highest (8.54 mm). Downward mobility showed reduced CT compared to stable high SEP (diff: 0.20 mm, *P* < .05), but no significant difference from upward mobility.

Supplemental Table 2 provides mean GM volumes, standard deviations, ANCOVA *F* values, and both uncorrected and FDR-corrected *P*-values for each social mobility group. Cortical thickness data are presented in Supplemental Table 3.

### Downward mobility negatively correlated with GM volume, not with thickness and global cognition

Table 3 presents estimations of DRMs for global brain structural and cognition outcomes. When looking at the independent role of each direction of mobility or distance, we found downward mobility specifically related to declined global GM volume, but not on cortical thickness. In contrast, neither mobility directions nor the changing status related to global cognition.

**Table 3:** Point estimate from diagonal reference models of association between social mobility and global brain cognitive outcomes, Whitehall II imaging study (*N* = 771)

	Grey matter volume (cm^3^)	Cortical thickness (mm)	Global cognition^a^
	Beta [95% CI]	Beta [95% CI]	Beta [95% CI]
Constant^b^	**509.657 [478.514, 540.800]** ^***^	**2.413 [2.372, 2.455]** ^***^	**28.967 [28.576, 29.357]** ^***^
Upward mobility	−5.483 [−18.544, 7.577]	−0.001 [−0.037, 0.035]	−0.321 [−0.944, 0.302]
Downward mobility	**−14.211 [−27.867, −0.554]** ^*^	−0.024 [−0.054, 0.006]	0.001 [−0.517, 0.517]
Mobility distance	2.666 [−2.908, 8.241]	0.006 [−0.007, 0.019]	0.152 [−0.079, 0.383]
Stable low SEP	**750.326 [701.171, 799.482]** ^**^	**2.715 [2.607, 2.8241]** ^**^	**33.659 [31.774, 35.543]** ^***^
Stable high SEP	**757.644 [710.332, 804.956]** ^**^	**2.758 [2.656, 2.861]** ^**^	**34.294 [32.441, 36.146]** ^***^
Age	**−2.157 [−3.430, −2.082]** ^**^	**−0.005 [−0.006, −0.003]** ^**^	**−0.083 [−0.109, −0.0563]** ^***^
Female	**−48.379 [−57.634, −39.106]** ^**^	0.015 [−0.006, 0.019]	**0.445 [0.087, 0.805]** ^**^
Weight on SEP origin	0.012	0.371	0.074
Weight on SEP destination	0.987	0.628	0.926
Goodness of fit			
AIC	5283.934	1050.979	1723.086
BIC	5317.919	1012.747	1829.665

*Notes*: AIC = Akaike information criterion; BIC = Bayesian information criterion

a Mini Mental State Examination Score at Phase 12 (2015–17)

b Constant indicates average value for female at mean age

Bold cell indicate statistical significance at ^*^P < .05, ^**^P < .01, ^***^P < .001

Meanwhile, being in a stable high SEP commonly indicates better performance in GM volume, cortical thickness and global cognition compared to stable low SEP. Females had smaller GM volume but slightly better global cognition score. The weights of origin and destination give the relative influence of father’s and individual mid-life SEP, with a higher relevance of destination weight, though not reaching a significant level.

In the sensitivity analysis, being mobile (versus stability) or educational mobility yielded insignificant results. Inclusion of other socio-lifestyle covariates slightly improved model fitness (Supplemental Table 4, smaller Akaike information criterion and Bayesian information criterion indicators). Other than gender and age, we found higher earlier fluid intelligence related to larger GM volumes (β = 1.366, *P* < .05) and global cognition (β = 0.043, *P* < .05). In addition, obesity served as a significant risk factor for global cognition (β = −0.475, *P* < .05).

### Social mobility did not predict changes in global cognition over time

**Figure 2:**
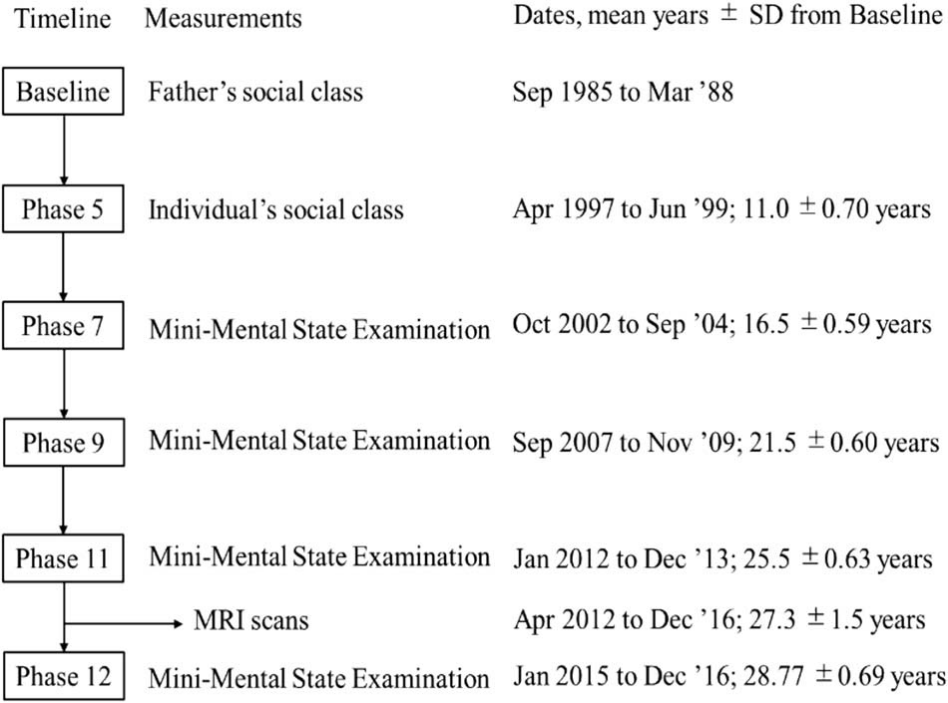
Timeline of MRI scan and global cognition evaluation.

Supplemental Table 5 presents estimations of linear mixed models verifying the interaction between mobility and time, with Model 2, adjusted for global brain structure and disease covariates, showing the best fit. Figure 2 provides a timeline of each evaluation of global cognition, with in total >28.7 years of follow-up from the original Whitehall Study. Our analysis, based on an average of 3.8 evaluations per respondent, found no significant relationship of social mobility (in either direction) on changes in later-life global cognition over time. We only observed that stable low SEP indicated the lowest initial score (Model 2: β = −3.53; 95% CI: −5.97 to −1.08, ref. stable high).

## Discussion

This study is the first, to our knowledge, to use whole brain analysis with diagonal reference models to describe the relationship between intergenerational social mobility and brain cognitive health in older life. We found that social mobility influences a wide range of grey matter volumes compared to cortical thickness at the whole brain level. Downward mobility is associated with the worst neurodegenerative status. However, despite these structural variations, the relationship of mobility and later-life global cognition appears to be minimal.

When parcellating the brain into multiple regions, we found that the regions showing the most significant structural variation were concentrated in the frontal and limbic lobes. The superior frontal gyrus has crucial influence on early executive function development [6, 35, 36], as well as on maintaining executive function as participants age [37]. The observed volumetric reduction in downward mobility experience may emphasise regional vulnerability in neuronal damage and disruption of neurogenesis in a particular mobile direction. Additionally, the posterior insular region’s reduction in volume suggests a possible link to the adverse influence of extended stress, as the insula region is involved in socio-emotional processing [38] and shows common volumetric reduction in depressive symptoms and early cognitive impairment, as highlighted by a recent meta-analysis [39]. Regional disturbance of the insula could be indirect evidence supporting the ‘stress hypothesis’, which suggests that extended levels of stress, financial disadvantage and hardships due to mobility can adversely affect brain regions [40]. The hippocampus, another critical region for long-term memory and social emotion [41], also showed the smallest volumes among the downward mobility group (left hippocampus *F* = 3.57, *P* < .02, right hippocampus *F* = 2.25, *P* = .55; Supplemental Table 2), aligning with previous regional imaging studies focusing on mobility and atrophy rate [21]. Taken together, the variations across different regions may reflect inefficiencies in daily cognitive and social functioning and increase the risk of further decline.

Our DRM approach confirmed an independent and negative association of downward mobility on grey matter volume, regardless of SEP origin and destination or the distance in status changes. In total, there were 2.3% and 3.9% smaller grey matter volumes in downward mobility compared to upward mobility and stable high SEP, respectively. Given that healthy adults experience atrophy in regional brain grey matter volume at a rate of 0.5% to 1.0% per year [42, 43], such findings may indicate an acceleration of normal brain degeneration by up to 3 years.

In contrast to brain structure, neither directions nor distances in status changes related to global cognition. Likewise, the recent report by De Looze *et al*. also found no mobility-specific relationship with MMSE-evaluated global cognition [20]. We consider three possible explanations. First, the influence of mobility on global cognition may still be marginal and not yet observable. It is important to note that brain grey matter volume atrophy begins years before cognitive decline [44], and mental stress (induced by mobility) accelerates neuron loss as individuals age and faster in regions responsible for memory [41]. As such, the estimated decline in GM regions could be early physiological markers of cognitive decline. Second is the possible selection bias. The high inclusion of upward mobility (535 out of 771 participants) and the high global cognition scores in the MRI scan (mean = 28.5, SD = 1.5) point to a relatively ‘better off’ population in our examination. This issue was also faced by the De Looze team when studying high educational and income populations. Empirical studies replicated that the improvements in healthier lifestyles and cognition of upward mobility is larger in populations with more socially disadvantaged backgrounds, such as ethnic minorities and immigrants [45, 46]. Thus, we could assume a larger effect size of mobility under a generally diverse population. Lastly, the low inclusion of stable low SEP may limit the statistical power in detecting the mobility associations. However, it is challenging to determine which direction of mobility would be more underestimated.

### Limitations and strengths

We acknowledge several crucial limitations. First, similarly to selection bias as prementioned (lacking diversity) is the possible misclassification. The proportion for skilled manual, semi-skilled and unskilled occupations is <5% (Supplemental Table 1) in our sample. In comparison, some skilled manual occupations such as electricians are highly socially famed with rising salaries in recent decades [47]. The stable low group included in the present sample may be relative to a ‘stable middle SEP’ in a general population. Thus, we considered an overall underestimation on the association of mobility with our included outcomes. Second, other psycho-social confounding factors, such as anxiety, sense of belongingness and mental insecurity, are commonly brought about by mobility in either direction. Without a detailed examination of psychological changes, we have limited ability to clarify endogeneity issues. Third, our definition of intergenerational mobility based on two timepoints was not representative of mobility trajectory over time; a further question that needs to be answered is, at what time point in one’s life course that mobility from original SEP would be beneficial or maladaptive to individual personal brain cognitive status? Finally, as MMSE is usually used as a clinical evaluation of dementia, it could have little power to detect early deviations among relatively healthy populations. A future analysis would benefit from including a diverse evaluation of multiple cognitive domains such as executive function and processing speed.

Nevertheless, we have strengths in a well-designed cohort that incorporates an automatic standardised imaging pipeline to process brain degeneration at the whole brain level, thus avoiding ignorance of relevant regions. Furthermore, with the confirmatory steps by DRMs, we are able to tell the distinct pathways from mobility. We additionally have strength in a longitudinal examination of cognitive measures by considering a wide range of socio-lifestyle covariates.

### Clinical and policy implications

We noted that grey matter volume, both regional and global measures, is more sensitive to mobility differences than cortical thickness. A recent imaging study observed similar results, showing that volume consistently reflects disadvantages in education and occupational variables across different countries [48]. Future neuroimaging studies may consider comparing grey matter volume with other structural measures (e.g. grey matter area, gyrification, and integrity between grey matter and white matter) to understand the socio-environmental burden at the molecular level. More importantly, these measures could be implemented into future prevention studies as precise evaluation proxies. Additionally, we highlight individuals’ vulnerability to socioeconomic surroundings, noting that both times of adverse exposure and changes across one’s life course produce distinct risks. Policymakers may need to consider life-course dynamics to identify high-risk subgroups more precisely and maximise prevention power on future cognitive disorders.

## Conclusion

While ‘rising from rags’ may not be beneficial, this study highlights the hindering cognitive risk of ‘falling from grace’, indicating an advanced level of brain degeneration in older life if one experiences downward mobility.

## Supplementary Material

aa-24-0322-File002_afae221
