# Supplementary material for: Influence of intergenerational social mobility on brain structure and global cognition: findings from the Whitehall II study across 20 years
Source: Age Ageing. 2024 Oct 12;53(10):afae221. doi: 10.1093/ageing/afae221 (PMC11470806; doi:10.1093/ageing/afae221)
Supplement: aa-24-0322-File002_afae221 [file aa-24-0322-file002_afae221.docx]

**Title.** Influence of Intergenerational Social Mobility On Brain Structure and Global Cognition: Findings From the Whitehall II Study Across 20 Years

Supplementary Text

Supplemental Table 1. Participants' distribution in the Registrar General's Social Classes scheme.

Supplemental Table 2. Regions of gray matter volume related to social mobility.

Supplemental Table 3. Regions of cortical thickness related to social mobility.

Supplemental Table 4. Association of social mobility and brain cognitie outcomes in full adjusted diagonal reference models. (N= 771)

Supplemental Table 5. Linear mixed models estimation on social mobility and cognitive change during follow-up , Whitehall II Study. (N= 771)

**Supplementary Text**

*MRI Acquisition and Preprocessing*

MRI data were acquired using one of two scanners: a 3T Siemens Magnetom Verio scanner (Erlangen, Germany) (n=552) or a 3T Siemens Magnetom Prisma scanner (Erlangen, Germany) (n=223) at the FMRIB Centre in the Wellcome Centre for Integrative Neuroimaging (WIN), Oxford. T1-weighted images were acquired using a Magnetization Prepared Rapid Acquisition Gradient Echo (MPRAGE) sequence (1mm^3^, Repetition Time [TR] = 2530ms, Time to Echo [TE] = 1.79/3.65/5.51/7.37ms) on the Verio scanner and a closely matched MPRAGE sequence on the Prisma scanner (1mm^3^, TR=1900ms, TE=3.97ms). T1 images were preprocessed using automated procedures in the Computational Anatomy Toolbox 12 (CAT12; Jena University Hospital, Germany; <http://www.neuro.uni-jena.de/cat/>). Initially, the gray matter (GM), white matter (WM), and cerebrospinal fluid (CSF) were segmented using Tissue Probability Maps to assign each voxel to its most likely tissue type and subsequent segmentation. Next, the GM and WM images were spatially normalized to the Montreal Neurological Institute (MNI) space by correcting the differences in the subjects’ head classs or orientation during scanning for the global brain shape. The image intensity of each voxel was modulated by Jacobian determinants and smoothed by convolving them with an 8-mm full width at half-maximum isotropic Gaussian kernel to construct GM volume-based maps at the whole-brain level. The thickness map was obtained in two steps after the spatial registration of GM and WM. The first step was to reconstruct the central surface, during which partial volume information, sulcal blurring, and sulcal asymmetries were allowed and then repaired using spherical harmonics to correct topological defects. Subsequently, the Freesurfer ‘FsAverage’ template was registered and smoothed by convolving them with a 15-mm full width at a half-maximum isotropic Gaussian kernel. This resulted in surface-based thickness maps at the whole-brain level. Finally, the value of thickness was calculated by estimating the distance between the inner surfaces (the boundary between the GM and WM) and outer surfaces (the boundary between the GM and CSF)[1].

*Covariates*

Sociodemographic data including age, sex, and marital status (married/cohabiting versus others) were based on self-report at the Imaging Sub-Study. Lifestyle disorders included obesity (WHO criteria, Body Mass Index ≥ 30[4]), depression classified by the Center for Epidemiologic Studies Depression Scale (CES-D; ≥ 16 scores as cut-off), and hypertension classified by the JN7 guideline (systolic blood pressure ≥ 140 mmHg or diastolic blood pressure ≥ 90 mmHg) [5]. Self-reported diabetes (yes/no) and social activity (10 leisure activity items i.e., visiting friends, gardening during the past 12 months, 0-3 scale indicating never to weekly) were derived from phase 11 for absence at the imaging Sub-Study. Fluid intelligence prior to MMSE evaluation (collected in phase 5: 1997–1999) was assessed using the Alice Heim 4-I test (AH4) composed of a series of verbal and mathematical reasoning items.

References:

**1**. Yotter RA, Dahnke R, Thompson PM, Gaser C. Topological correction of brain surface meshes using spherical harmonics. Hum Brain Mapp 2011; 32: 1109–1124.

**2**. Stewart AL, Mills KM, King AC, Haskell WL, Gillis D, Ritter PL. CHAMPS Physical Activity Questionnaire for Older Adults: outcomes for interventions. Med Sci Sports Exerc 2001; 33: 1126–1141.

**3**. Buysse DJ, Reynolds CF, Monk TH, Berman SR, Kupfer DJ. The Pittsburgh Sleep Quality Index: a new instrument for psychiatric practice and research. Psychiatry Res 1989; 28: 193–213.

**4**. James PT, Leach R, Kalamara E, Shayeghi M. The Worldwide Obesity Epidemic. Obes Res 2001; 9: 228S-233S.

**5**. Chobanian A v., Bakris GL, Black HR *et al.* The Seventh Report of the Joint National Committee on Prevention, Detection, Evaluation, and Treatment of High Blood Pressure: the JNC 7 report. JAMA 2003; 289: 2560–2572.

| Supplemental Table 1. Participants' distribution in the Registrar General's Social Classes scheme. | | | |
| --- | --- | --- | --- |
| Class | N (Cum) | Context | Job title |
| Leve 1 | 0 | Unskilled occupations | Labourers, cleaners. |
| Leve 2 | 11 (1.43) | Semi-skilled occupations | Security guards, care assistants. |
| Leve 3 | 17 (2.20) | Skilled manual occupations | Supervisors, plumbers, electricans. |
| Leve 4 | 49 (6.36) | Skilled non-manual occupations | Clerks, cashiers, retail staff. |
| Leve 5 | 588 (76.26) | Intermediate occupations | Manager, school teacher, journalists. |
| Leve 6 | 106 (13.75) | Professional occupations | Doctors, lawyers, accountats, engineers. |
|  |  |  |  |

| Supplemental Table 2. Regions of gray matter volume related to social mobility. | | | | | | | |
| --- | --- | --- | --- | --- | --- | --- | --- |
|  | Stable High | Upward Mobility | Stable Low | Downward Mobility | F | *P*_uncorr._ | *P*_fdr_ |
| **Left SFG superior frontal gyrus (mean (SD))** | 11.97 (1.49) | 11.71 (1.32) | 11.87 (1.61) | 11.25 (1.47) | 8.677 | 0.000011 | 0.000014 |
| **Left PIns posterior insula (mean (SD))** | 1.84 (0.21) | 1.81 (0.19) | 1.88 (0.20) | 1.75 (0.22) | 6.583 | 0.000214 | 0.000268 |
| **Left FuG fusiform gyrus (mean (SD))** | 6.05 (0.77) | 5.99 (0.62) | 6.08 (0.58) | 5.78 (0.71) | 6.448 | 0.000259 | 0.000323 |
| **Right PIns posterior insula (mean (SD))** | 1.97 (0.22) | 1.94 (0.20) | 1.94 (0.22) | 1.87 (0.22) | 6.360 | 0.000293 | 0.000366 |
| **Left MPrG precentral gyrus medial segment (mean (SD))** | 1.93 (0.30) | 1.87 (0.29) | 1.79 (0.26) | 1.79 (0.28) | 5.588 | 0.000856 | 0.001071 |
| **Left PP planum polare (mean (SD))** | 1.66 (0.21) | 1.63 (0.19) | 1.73 (0.22) | 1.59 (0.24) | 5.272 | 0.001329 | 0.001661 |
| **Left MCgG middle cingulate gyrus (mean (SD))** | 3.44 (0.42) | 3.40 (0.38) | 3.32 (0.39) | 3.27 (0.37) | 5.261 | 0.001348 | 0.001685 |
| **Right AOrG anterior orbital gyrus (mean (SD))** | 1.57 (0.20) | 1.55 (0.19) | 1.48 (0.17) | 1.50 (0.19) | 5.123 | 0.001633 | 0.002041 |
| **Left SMC supplementary motor cortex (mean (SD))** | 4.39 (0.59) | 4.33 (0.51) | 4.15 (0.45) | 4.18 (0.52) | 5.082 | 0.001728 | 0.002160 |
| **Left PHG parahippocampal gyrus (mean (SD))** | 2.83 (0.34) | 2.77 (0.29) | 2.83 (0.26) | 2.72 (0.32) | 5.037 | 0.001839 | 0.002298 |
| **Right PHG parahippocampal gyrus (mean (SD))** | 2.75 (0.31) | 2.69 (0.27) | 2.72 (0.23) | 2.65 (0.31) | 4.995 | 0.001950 | 0.002438 |
| **Right PP planum polare (mean (SD))** | 1.51 (0.20) | 1.48 (0.17) | 1.55 (0.19) | 1.44 (0.18) | 4.975 | 0.002003 | 0.002504 |
| **Left Basal Forebrain (mean (SD))** | 0.35 (0.05) | 0.35 (0.05) | 0.36 (0.06) | 0.33 (0.05) | 4.905 | 0.002209 | 0.002762 |
| **Left POrG posterior orbital gyrus (mean (SD))** | 2.49 (0.35) | 2.45 (0.31) | 2.50 (0.28) | 2.36 (0.32) | 4.757 | 0.002710 | 0.003388 |
| **Right Accumbens Area (mean (SD))** | 0.37 (0.05) | 0.36 (0.04) | 0.36 (0.04) | 0.35 (0.04) | 4.730 | 0.002814 | 0.003517 |
| **Left ACgG anterior cingulate gyrus (mean (SD))** | 3.82 (0.51) | 3.86 (0.50) | 3.74 (0.49) | 3.70 (0.47) | 4.686 | 0.002989 | 0.003736 |
| **Left MTG middle temporal gyrus (mean (SD))** | 11.87 (1.44) | 11.70 (1.30) | 11.87 (1.12) | 11.39 (1.28) | 4.637 | 0.003197 | 0.003996 |
| **Left PCu precuneus (mean (SD))** | 9.02 (1.09) | 8.87 (1.01) | 8.74 (0.80) | 8.62 (1.14) | 4.478 | 0.003981 | 0.004976 |
| **Right MOrG medial orbital gyrus (mean (SD))** | 3.57 (0.39) | 3.56 (0.37) | 3.48 (0.42) | 3.44 (0.36) | 4.465 | 0.004054 | 0.005067 |
| **Left STG superior temporal gyrus (mean (SD))** | 5.86 (0.71) | 5.74 (0.65) | 5.70 (0.54) | 5.60 (0.76) | 4.271 | 0.005297 | 0.006621 |
| **Left LiG lingual gyrus (mean (SD))** | 6.04 (0.81) | 5.90 (0.72) | 6.02 (0.76) | 5.76 (0.77) | 4.245 | 0.005492 | 0.006865 |
| **Right OCP occipital pole (mean (SD))** | 2.61 (0.55) | 2.70 (0.51) | 2.38 (0.39) | 2.60 (0.50) | 4.200 | 0.005840 | 0.007300 |
| **Right SMC supplementary motor cortex (mean (SD))** | 4.21 (0.61) | 4.18 (0.51) | 4.17 (0.49) | 4.00 (0.58) | 4.114 | 0.006574 | 0.008217 |
| **Right GRe gyrus rectus (mean (SD))** | 1.86 (0.23) | 1.84 (0.23) | 1.79 (0.21) | 1.78 (0.22) | 3.682 | 0.011864 | 0.011864 |
| **Left Accumbens Area (mean (SD))** | 0.40 (0.05) | 0.39 (0.05) | 0.38 (0.04) | 0.38 (0.06) | 3.807 | 0.010001 | 0.012501 |
| **Right ITG inferior temporal gyrus (mean (SD))** | 10.47 (1.32) | 10.39 (1.14) | 10.52 (1.34) | 10.10 (1.28) | 3.717 | 0.011316 | 0.014145 |
| **Right TMP temporal pole (mean (SD))** | 8.26 (1.04) | 8.12 (0.92) | 7.88 (0.79) | 7.93 (1.05) | 3.710 | 0.011422 | 0.014278 |
| **Left MOrG medial orbital gyrus (mean (SD))** | 3.59 (0.38) | 3.60 (0.39) | 3.51 (0.35) | 3.49 (0.39) | 3.702 | 0.011553 | 0.014441 |
| **Left AIns anterior insula (mean (SD))** | 3.75 (0.38) | 3.70 (0.36) | 3.78 (0.51) | 3.62 (0.44) | 3.694 | 0.011683 | 0.014603 |
| **Left Hippocampus (mean (SD))** | 2.96 (0.31) | 2.90 (0.30) | 2.97 (0.32) | 2.87 (0.34) | 3.570 | 0.013817 | 0.017271 |
| **Right AIns anterior insula (mean (SD))** | 3.76 (0.37) | 3.71 (0.36) | 3.78 (0.43) | 3.64 (0.45) | 3.521 | 0.014786 | 0.018483 |
| **Left Putamen (mean (SD))** | 3.50 (0.47) | 3.45 (0.45) | 3.20 (0.47) | 3.36 (0.48) | 3.713 | 0.011381 | 0.018968 |
| **Right Amygdala (mean (SD))** | 0.86 (0.08) | 0.85 (0.09) | 0.84 (0.07) | 0.83 (0.09) | 3.480 | 0.015629 | 0.019537 |
| **Left PCgG posterior cingulate gyrus (mean (SD))** | 3.52 (0.41) | 3.45 (0.41) | 3.45 (0.41) | 3.39 (0.43) | 3.378 | 0.017943 | 0.022429 |
| **Right Ent entorhinal area (mean (SD))** | 1.91 (0.24) | 1.90 (0.23) | 1.88 (0.21) | 1.83 (0.23) | 3.357 | 0.018463 | 0.023079 |
| **Right MTG middle temporal gyrus (mean (SD))** | 12.50 (1.50) | 12.30 (1.30) | 12.41 (1.18) | 12.12 (1.36) | 3.343 | 0.018809 | 0.023511 |
| **Left PrG precentral gyrus (mean (SD))** | 9.16 (1.32) | 9.05 (1.16) | 9.15 (1.03) | 8.72 (1.12) | 3.274 | 0.020654 | 0.025817 |
| **Right STG superior temporal gyrus (mean (SD))** | 6.08 (0.81) | 5.96 (0.67) | 6.07 (0.59) | 5.86 (0.74) | 3.158 | 0.024168 | 0.030210 |
| **Right PrG precentral gyrus (mean (SD))** | 9.02 (1.20) | 8.90 (1.11) | 8.80 (0.95) | 8.62 (0.97) | 3.155 | 0.024287 | 0.030359 |
| **Right SPL superior parietal lobule (mean (SD))** | 8.34 (0.99) | 8.17 (0.90) | 7.92 (1.13) | 8.08 (1.04) | 3.096 | 0.026286 | 0.032858 |
| **Left ITG inferior temporal gyrus (mean (SD))** | 10.07 (1.35) | 10.01 (1.12) | 10.14 (1.03) | 9.76 (1.11) | 3.022 | 0.029044 | 0.036304 |
| **Right MFC medial frontal cortex (mean (SD))** | 1.50 (0.23) | 1.49 (0.21) | 1.42 (0.22) | 1.44 (0.18) | 2.969 | 0.031185 | 0.038982 |
| **Right Putamen (mean (SD))** | 3.57 (0.41) | 3.50 (0.41) | 3.34 (0.29) | 3.44 (0.41) | 3.177 | 0.023575 | 0.039292 |
| **Left AOrG anterior orbital gyrus (mean (SD))** | 1.41 (0.19) | 1.43 (0.17) | 1.36 (0.13) | 1.39 (0.19) | 2.893 | 0.034537 | 0.043171 |
| **Right MFG middle frontal gyrus (mean (SD))** | 15.04 (1.86) | 14.90 (1.79) | 14.84 (1.78) | 14.51 (1.64) | 2.873 | 0.035469 | 0.044336 |
| **Left OFuG occipital fusiform gyrus (mean (SD))** | 3.20 (0.44) | 3.19 (0.41) | 3.33 (0.45) | 3.11 (0.42) | 2.829 | 0.037659 | 0.047074 |
| **Right OrIFG orbital part of the inferior frontal gyrus (mean (SD))** | 1.19 (0.18) | 1.16 (0.17) | 1.12 (0.20) | 1.14 (0.15) | 2.816 | 0.038308 | 0.047885 |
| **Right FuG fusiform gyrus (mean (SD))** | 6.40 (0.79) | 6.34 (0.69) | 6.39 (0.54) | 6.21 (0.76) | 2.647 | 0.047984 | 0.047984 |
| **Right SCA subcallosal area (mean (SD))** | 1.09 (0.15) | 1.09 (0.15) | 1.08 (0.15) | 1.05 (0.13) | 2.782 | 0.040067 | 0.050084 |
| Right Cerebellum Exterior (mean (SD)) | 40.04 (4.71) | 39.33 (4.26) | 38.99 (3.81) | 38.67 (4.29) | 2.585 | 0.052169 | 0.052169 |
| Right LOrG lateral orbital gyrus (mean (SD)) | 1.82 (0.25) | 1.79 (0.22) | 1.76 (0.24) | 1.75 (0.21) | 2.537 | 0.055611 | 0.055611 |
| Left MFG middle frontal gyrus (mean (SD)) | 14.94 (1.86) | 14.82 (1.80) | 14.67 (1.61) | 14.41 (2.00) | 2.621 | 0.049688 | 0.062110 |
| Left Cerebellum Exterior (mean (SD)) | 39.87 (4.71) | 39.22 (4.27) | 38.72 (3.57) | 38.51 (4.38) | 2.452 | 0.062253 | 0.062253 |
| Left LOrG lateral orbital gyrus (mean (SD)) | 1.91 (0.27) | 1.88 (0.22) | 1.89 (0.20) | 1.84 (0.24) | 2.436 | 0.063531 | 0.063531 |
| Right POrG posterior orbital gyrus (mean (SD)) | 2.36 (0.32) | 2.33 (0.29) | 2.27 (0.26) | 2.27 (0.30) | 2.575 | 0.052868 | 0.066085 |
| Left Amygdala (mean (SD)) | 0.87 (0.10) | 0.86 (0.09) | 0.85 (0.11) | 0.84 (0.09) | 2.390 | 0.067493 | 0.067493 |
| Right Hippocampus (mean (SD)) | 3.17 (0.31) | 3.12 (0.32) | 3.13 (0.27) | 3.08 (0.35) | 2.545 | 0.055026 | 0.068783 |
| 3rd Ventricle (mean (SD)) | 0.05 (0.03) | 0.05 (0.03) | 0.07 (0.06) | 0.05 (0.03) | 2.737 | 0.042556 | 0.070927 |
| Right MSFG superior frontal gyrus medial segment (mean (SD)) | 6.40 (0.90) | 6.35 (0.87) | 6.27 (0.86) | 6.15 (0.65) | 2.503 | 0.058111 | 0.072639 |
| Cerebellar Vermal Lobules VI-VII (mean (SD)) | 1.62 (0.20) | 1.58 (0.20) | 1.52 (0.14) | 1.57 (0.20) | 2.423 | 0.064622 | 0.080777 |
| Left MSFG superior frontal gyrus medial segment (mean (SD)) | 5.32 (0.60) | 5.30 (0.55) | 5.24 (0.50) | 5.16 (0.57) | 2.415 | 0.065313 | 0.081641 |
| Left TMP temporal pole (mean (SD)) | 8.13 (1.03) | 8.03 (0.95) | 7.88 (1.13) | 7.83 (1.09) | 2.412 | 0.065591 | 0.081989 |
| Left SPL superior parietal lobule (mean (SD)) | 8.76 (1.05) | 8.77 (0.95) | 8.41 (0.92) | 8.59 (0.97) | 2.241 | 0.082155 | 0.082155 |
| Right Cun cuneus (mean (SD)) | 3.69 (0.57) | 3.65 (0.49) | 3.45 (0.54) | 3.57 (0.49) | 2.405 | 0.066208 | 0.082760 |
| Cerebellar Vermal Lobules I-V (mean (SD)) | 3.60 (0.47) | 3.55 (0.44) | 3.32 (0.34) | 3.53 (0.45) | 2.609 | 0.050539 | 0.084232 |
| Right OpIFG opercular part of the inferior frontal gyrus (mean (SD)) | 2.54 (0.32) | 2.60 (0.34) | 2.63 (0.44) | 2.53 (0.37) | 2.383 | 0.068171 | 0.085214 |
| Right PCu precuneus (mean (SD)) | 9.27 (1.09) | 9.15 (0.99) | 8.96 (0.62) | 9.03 (1.10) | 2.168 | 0.090425 | 0.090425 |
| Left Cun cuneus (mean (SD)) | 3.32 (0.53) | 3.32 (0.48) | 3.07 (0.49) | 3.26 (0.44) | 2.253 | 0.080915 | 0.101144 |
| Right PCgG posterior cingulate gyrus (mean (SD)) | 3.17 (0.38) | 3.14 (0.36) | 3.10 (0.35) | 3.08 (0.37) | 2.075 | 0.102051 | 0.102051 |
| Right FO frontal operculum (mean (SD)) | 1.59 (0.19) | 1.59 (0.20) | 1.64 (0.25) | 1.54 (0.21) | 2.234 | 0.082946 | 0.103682 |
| Right OFuG occipital fusiform gyrus (mean (SD)) | 3.02 (0.40) | 3.02 (0.39) | 3.13 (0.35) | 2.95 (0.38) | 2.039 | 0.106978 | 0.106978 |
| Right Basal Forebrain (mean (SD)) | 0.36 (0.04) | 0.36 (0.04) | 0.36 (0.04) | 0.35 (0.04) | 2.210 | 0.085633 | 0.107042 |
| Right FRP frontal pole (mean (SD)) | 3.21 (0.45) | 3.24 (0.47) | 3.19 (0.43) | 3.13 (0.43) | 2.189 | 0.088015 | 0.110019 |
| Left SCA subcallosal area (mean (SD)) | 1.13 (0.16) | 1.14 (0.16) | 1.13 (0.16) | 1.10 (0.17) | 2.173 | 0.089807 | 0.112259 |
| Left MOG middle occipital gyrus (mean (SD)) | 4.85 (0.68) | 4.83 (0.58) | 4.92 (0.55) | 4.70 (0.57) | 2.168 | 0.090425 | 0.113031 |
| Left MPoG postcentral gyrus medial segment (mean (SD)) | 0.75 (0.14) | 0.73 (0.15) | 0.70 (0.11) | 0.71 (0.16) | 2.135 | 0.094439 | 0.118049 |
| Right MCgG middle cingulate gyrus (mean (SD)) | 3.57 (0.41) | 3.54 (0.42) | 3.45 (0.38) | 3.46 (0.46) | 2.125 | 0.095601 | 0.119501 |
| Left FRP frontal pole (mean (SD)) | 2.89 (0.46) | 2.91 (0.40) | 2.93 (0.40) | 2.80 (0.40) | 2.335 | 0.072645 | 0.121075 |
| Left PT planum temporale (mean (SD)) | 1.73 (0.31) | 1.69 (0.26) | 1.73 (0.29) | 1.65 (0.32) | 2.082 | 0.101138 | 0.126422 |
| Right MOG middle occipital gyrus (mean (SD)) | 4.03 (0.57) | 4.06 (0.53) | 4.07 (0.56) | 3.93 (0.60) | 2.046 | 0.106084 | 0.132605 |
| Right MPrG precentral gyrus medial segment (mean (SD)) | 1.93 (0.32) | 1.89 (0.29) | 1.89 (0.32) | 1.84 (0.29) | 2.000 | 0.112512 | 0.140640 |
| Right Caudate (mean (SD)) | 2.85 (0.41) | 2.79 (0.39) | 2.75 (0.39) | 2.74 (0.36) | 2.176 | 0.089512 | 0.149187 |
| Left OpIFG opercular part of the inferior frontal gyrus (mean (SD)) | 2.41 (0.34) | 2.40 (0.32) | 2.46 (0.31) | 2.33 (0.32) | 1.775 | 0.150423 | 0.188028 |
| Right SFG superior frontal gyrus (mean (SD)) | 11.77 (1.42) | 11.65 (1.41) | 11.78 (1.31) | 11.45 (1.40) | 1.582 | 0.192200 | 0.192200 |
| Left Caudate (mean (SD)) | 2.71 (0.38) | 2.66 (0.36) | 2.58 (0.41) | 2.63 (0.34) | 1.623 | 0.182622 | 0.212652 |
| Right SMG supramarginal gyrus (mean (SD)) | 6.53 (0.92) | 6.43 (0.77) | 6.52 (0.96) | 6.36 (0.81) | 1.446 | 0.228000 | 0.228000 |
| Left Ent entorhinal area (mean (SD)) | 1.79 (0.22) | 1.77 (0.21) | 1.74 (0.28) | 1.74 (0.23) | 1.622 | 0.182903 | 0.228629 |
| Left TTG transverse temporal gyrus (mean (SD)) | 1.14 (0.23) | 1.11 (0.19) | 1.11 (0.13) | 1.09 (0.21) | 1.593 | 0.189632 | 0.237039 |
| Right PoG postcentral gyrus (mean (SD)) | 7.51 (1.03) | 7.52 (0.98) | 7.56 (0.66) | 7.30 (0.91) | 1.582 | 0.192329 | 0.240411 |
| Left CO central operculum (mean (SD)) | 3.28 (0.44) | 3.26 (0.41) | 3.36 (0.35) | 3.20 (0.45) | 1.365 | 0.252298 | 0.252298 |
| Right PO parietal operculum (mean (SD)) | 1.60 (0.28) | 1.59 (0.25) | 1.68 (0.18) | 1.56 (0.26) | 1.453 | 0.226064 | 0.282580 |
| 4th Ventricle (mean (SD)) | 0.16 (0.05) | 0.17 (0.06) | 0.16 (0.04) | 0.17 (0.05) | 1.679 | 0.170162 | 0.283603 |
| Left PO parietal operculum (mean (SD)) | 1.97 (0.31) | 1.96 (0.28) | 2.06 (0.26) | 1.94 (0.30) | 1.430 | 0.232582 | 0.290728 |
| Right ACgG anterior cingulate gyrus (mean (SD)) | 3.13 (0.48) | 3.17 (0.53) | 3.18 (0.52) | 3.06 (0.48) | 1.423 | 0.234721 | 0.293402 |
| Left OrIFG orbital part of the inferior frontal gyrus (mean (SD)) | 1.32 (0.18) | 1.29 (0.18) | 1.29 (0.17) | 1.30 (0.18) | 1.418 | 0.236160 | 0.295200 |
| Right LiG lingual gyrus (mean (SD)) | 6.45 (0.88) | 6.40 (0.73) | 6.40 (0.71) | 6.28 (0.69) | 1.228 | 0.298277 | 0.298277 |
| Left IOG inferior occipital gyrus (mean (SD)) | 5.20 (0.67) | 5.20 (0.65) | 5.30 (0.72) | 5.09 (0.68) | 1.401 | 0.241120 | 0.301400 |
| Left FO frontal operculum (mean (SD)) | 1.52 (0.20) | 1.51 (0.18) | 1.51 (0.21) | 1.48 (0.21) | 1.018 | 0.384137 | 0.384137 |
| Left OCP occipital pole (mean (SD)) | 2.89 (0.51) | 2.95 (0.55) | 2.91 (0.65) | 2.85 (0.55) | 1.201 | 0.308425 | 0.385532 |
| Left PoG postcentral gyrus (mean (SD)) | 8.43 (1.10) | 8.33 (1.03) | 8.51 (0.90) | 8.24 (1.02) | 1.173 | 0.318954 | 0.398693 |
| Left GRe gyrus rectus (mean (SD)) | 1.80 (0.22) | 1.79 (0.22) | 1.79 (0.18) | 1.76 (0.24) | 0.969 | 0.406998 | 0.406998 |
| Right PT planum temporale (mean (SD)) | 1.59 (0.25) | 1.57 (0.24) | 1.57 (0.17) | 1.54 (0.28) | 1.079 | 0.357018 | 0.446272 |
| Right Inf Lat Vent (mean (SD)) | 0.05 (0.02) | 0.05 (0.02) | 0.05 (0.02) | 0.05 (0.02) | 1.286 | 0.277902 | 0.463169 |
| Right SOG superior occipital gyrus (mean (SD)) | 3.15 (0.43) | 3.12 (0.39) | 3.04 (0.48) | 3.08 (0.44) | 0.820 | 0.483165 | 0.483940 |
| Left MFC medial frontal cortex (mean (SD)) | 1.51 (0.21) | 1.50 (0.21) | 1.51 (0.24) | 1.47 (0.19) | 0.802 | 0.492966 | 0.492966 |
| Right TrIFG triangular part of the inferior frontal gyrus (mean (SD)) | 2.82 (0.40) | 2.82 (0.37) | 2.84 (0.33) | 2.76 (0.34) | 0.809 | 0.488829 | 0.508736 |
| Right AnG angular gyrus (mean (SD)) | 8.31 (1.10) | 8.28 (0.97) | 8.30 (0.93) | 8.16 (1.00) | 0.716 | 0.542357 | 0.542357 |
| Right CO central operculum (mean (SD)) | 3.31 (0.45) | 3.31 (0.43) | 3.37 (0.39) | 3.26 (0.47) | 0.697 | 0.553847 | 0.553847 |
| Left Ventral DC (mean (SD)) | 0.85 (0.11) | 0.84 (0.11) | 0.86 (0.10) | 0.83 (0.13) | 0.685 | 0.561512 | 0.561512 |
| Right IOG inferior occipital gyrus (mean (SD)) | 5.17 (0.63) | 5.19 (0.64) | 5.10 (0.53) | 5.11 (0.71) | 0.663 | 0.574918 | 0.574918 |
| Left Pallidum (mean (SD)) | 0.25 (0.12) | 0.24 (0.12) | 0.26 (0.16) | 0.23 (0.10) | 0.848 | 0.467644 | 0.584554 |
| Right TTG transverse temporal gyrus (mean (SD)) | 0.96 (0.17) | 0.96 (0.15) | 0.96 (0.15) | 0.94 (0.15) | 0.641 | 0.588513 | 0.588513 |
| Right Ventral DC (mean (SD)) | 0.84 (0.11) | 0.83 (0.10) | 0.83 (0.11) | 0.82 (0.12) | 0.588 | 0.623299 | 0.623299 |
| Cerebellar Vermal Lobules VIII-X (mean (SD)) | 2.01 (0.31) | 2.01 (0.31) | 1.94 (0.24) | 1.98 (0.30) | 0.555 | 0.644668 | 0.644668 |
| Optic Chiasm (mean (SD)) | 0.10 (0.03) | 0.10 (0.03) | 0.11 (0.03) | 0.11 (0.03) | 0.469 | 0.704112 | 0.704112 |
| Left SMG supramarginal gyrus (mean (SD)) | 7.08 (0.86) | 7.09 (0.85) | 7.08 (0.67) | 6.99 (0.96) | 0.460 | 0.710533 | 0.710533 |
| Left AnG angular gyrus (mean (SD)) | 7.29 (0.95) | 7.23 (0.85) | 7.17 (0.76) | 7.19 (0.99) | 0.439 | 0.725182 | 0.725182 |
| Right Calc calcarine cortex (mean (SD)) | 2.28 (0.41) | 2.30 (0.37) | 2.24 (0.35) | 2.24 (0.31) | 0.641 | 0.589006 | 0.729196 |
| Left Inf Lat Vent (mean (SD)) | 0.02 (0.01) | 0.02 (0.01) | 0.03 (0.01) | 0.02 (0.01) | 0.593 | 0.619590 | 0.740375 |
| Left SOG superior occipital gyrus (mean (SD)) | 2.74 (0.37) | 2.73 (0.35) | 2.67 (0.30) | 2.69 (0.38) | 0.482 | 0.695004 | 0.742344 |
| Left Thalamus Proper (mean (SD)) | 4.47 (0.75) | 4.40 (0.80) | 4.52 (0.98) | 4.41 (0.95) | 0.382 | 0.766167 | 0.766167 |
| Right Pallidum (mean (SD)) | 0.26 (0.11) | 0.25 (0.12) | 0.26 (0.14) | 0.24 (0.11) | 0.352 | 0.787475 | 0.787475 |
| Left TrIFG triangular part of the inferior frontal gyrus (mean (SD)) | 2.87 (0.42) | 2.85 (0.37) | 2.80 (0.33) | 2.84 (0.39) | 0.325 | 0.807258 | 0.807258 |
| Right MPoG postcentral gyrus medial segment (mean (SD)) | 0.76 (0.16) | 0.75 (0.15) | 0.75 (0.14) | 0.75 (0.13) | 0.318 | 0.812473 | 0.812473 |
| Right Lateral Ventricle (mean (SD)) | 0.42 (0.13) | 0.43 (0.16) | 0.45 (0.18) | 0.41 (0.13) | 0.795 | 0.496689 | 0.827815 |
| Right Thalamus Proper (mean (SD)) | 4.55 (0.87) | 4.54 (0.83) | 4.41 (1.08) | 4.48 (0.88) | 0.277 | 0.842358 | 0.842358 |
| Left Lateral Ventricle (mean (SD)) | 0.43 (0.15) | 0.42 (0.16) | 0.45 (0.19) | 0.43 (0.16) | 0.193 | 0.901167 | 0.901167 |
| Left Calc calcarine cortex (mean (SD)) | 2.32 (0.40) | 2.30 (0.39) | 2.29 (0.43) | 2.29 (0.33) | 0.161 | 0.922562 | 0.952630 |
| Brain Stem (mean (SD)) | 1.20 (0.44) | 1.18 (0.46) | 1.21 (0.45) | 1.17 (0.44) | 0.076 | 0.973094 | 0.973094 |
| Note: bold fonts suggest P_fdr_<0.05 | | | | | | | |

| Supplemental Table 3. Regions of cortical thickness related to social mobility. | | | | | | | |
| --- | --- | --- | --- | --- | --- | --- | --- |
|  | Stable High | Upward Mobility | Stable Low | Downward Mobility | F | *P*_uncorr._ | *P*_fdr_ |
| **Right cuneus (mean (SD))** | 1.89 (0.16) | 1.87 (0.14) | 1.75 (0.16) | 1.84 (0.15) | 5.435 | 0.001 | 0.003 |
| **Right lateralorbitofrontal (mean (SD))** | 2.64 (0.13) | 2.63 (0.12) | 2.58 (0.18) | 2.59 (0.13) | 4.141 | 0.006 | 0.032 |
| **Left cuneus (mean (SD))** | 1.90 (0.15) | 1.87 (0.15) | 1.79 (0.15) | 1.88 (0.15) | 3.305 | 0.020 | 0.033 |
| **Left paracentral (mean (SD))** | 2.11 (0.22) | 2.08 (0.22) | 2.02 (0.22) | 2.03 (0.20) | 3.050 | 0.028 | 0.047 |
| Left caudalmiddlefrontal (mean (SD)) | 2.43 (0.14) | 2.41 (0.14) | 2.40 (0.16) | 2.37 (0.19) | 3.171 | 0.024 | 0.059 |
| Left precentral (mean (SD)) | 2.11 (0.19) | 2.07 (0.17) | 2.06 (0.15) | 2.04 (0.16) | 2.781 | 0.040 | 0.067 |
| Left lateralorbitofrontal (mean (SD)) | 2.62 (0.12) | 2.61 (0.12) | 2.58 (0.15) | 2.58 (0.11) | 2.607 | 0.051 | 0.084 |
| Left postcentral (mean (SD)) | 1.93 (0.14) | 1.91 (0.13) | 1.92 (0.16) | 1.88 (0.12) | 2.470 | 0.061 | 0.101 |
| Right precuneus (mean (SD)) | 2.34 (0.13) | 2.32 (0.11) | 2.30 (0.10) | 2.30 (0.11) | 2.468 | 0.061 | 0.102 |
| Right pericalcarine (mean (SD)) | 1.66 (0.18) | 1.63 (0.19) | 1.58 (0.21) | 1.60 (0.19) | 2.115 | 0.097 | 0.121 |
| Left lingual (mean (SD)) | 1.92 (0.14) | 1.90 (0.13) | 1.90 (0.17) | 1.87 (0.17) | 1.812 | 0.143 | 0.179 |
| Right superiortemporal (mean (SD)) | 2.69 (0.14) | 2.67 (0.13) | 2.67 (0.16) | 2.65 (0.14) | 2.029 | 0.108 | 0.181 |
| Left posteriorcingulate (mean (SD)) | 2.29 (0.14) | 2.28 (0.13) | 2.26 (0.17) | 2.25 (0.12) | 1.928 | 0.124 | 0.206 |
| Left pericalcarine (mean (SD)) | 1.67 (0.16) | 1.64 (0.18) | 1.59 (0.16) | 1.63 (0.16) | 1.904 | 0.127 | 0.212 |
| Right parahippocampal (mean (SD)) | 2.41 (0.18) | 2.39 (0.19) | 2.45 (0.21) | 2.36 (0.17) | 1.862 | 0.135 | 0.224 |
| Left entorhinal (mean (SD)) | 3.43 (0.27) | 3.42 (0.29) | 3.32 (0.45) | 3.37 (0.26) | 1.630 | 0.181 | 0.226 |
| Left bankssts (mean (SD)) | 2.47 (0.13) | 2.44 (0.13) | 2.42 (0.12) | 2.44 (0.14) | 1.854 | 0.136 | 0.226 |
| Left precuneus (mean (SD)) | 2.33 (0.13) | 2.31 (0.11) | 2.33 (0.12) | 2.30 (0.12) | 2.147 | 0.093 | 0.232 |
| Right lingual (mean (SD)) | 1.93 (0.13) | 1.92 (0.13) | 1.93 (0.17) | 1.89 (0.13) | 1.609 | 0.186 | 0.232 |
| Left inferiorparietal (mean (SD)) | 2.38 (0.12) | 2.36 (0.10) | 2.36 (0.12) | 2.35 (0.10) | 1.578 | 0.193 | 0.242 |
| Right lateraloccipital (mean (SD)) | 2.15 (0.13) | 2.13 (0.11) | 2.09 (0.13) | 2.12 (0.12) | 1.712 | 0.163 | 0.272 |
| Right parsorbitalis (mean (SD)) | 2.52 (0.14) | 2.51 (0.13) | 2.50 (0.23) | 2.48 (0.15) | 1.626 | 0.182 | 0.303 |
| Right superiorparietal (mean (SD)) | 2.16 (0.14) | 2.14 (0.12) | 2.14 (0.12) | 2.12 (0.12) | 1.620 | 0.183 | 0.306 |
| Left inferiortemporal (mean (SD)) | 2.65 (0.12) | 2.64 (0.11) | 2.66 (0.12) | 2.62 (0.12) | 1.183 | 0.315 | 0.315 |
| Right transversetemporal (mean (SD)) | 2.11 (0.27) | 2.07 (0.22) | 2.07 (0.30) | 2.04 (0.20) | 1.860 | 0.135 | 0.337 |
| Left middletemporal (mean (SD)) | 2.67 (0.12) | 2.65 (0.12) | 2.63 (0.12) | 2.65 (0.11) | 1.530 | 0.205 | 0.342 |
| Right insula (mean (SD)) | 3.07 (0.17) | 3.04 (0.19) | 2.99 (0.21) | 3.01 (0.19) | 1.848 | 0.137 | 0.343 |
| Left rostralmiddlefrontal (mean (SD)) | 2.31 (0.11) | 2.30 (0.11) | 2.28 (0.15) | 2.28 (0.11) | 1.494 | 0.215 | 0.344 |
| Left superiorfrontal (mean (SD)) | 2.54 (0.13) | 2.53 (0.12) | 2.51 (0.16) | 2.51 (0.11) | 1.289 | 0.277 | 0.346 |
| Left superiorparietal (mean (SD)) | 2.18 (0.13) | 2.16 (0.12) | 2.16 (0.12) | 2.14 (0.13) | 1.471 | 0.221 | 0.369 |
| Right inferiortemporal (mean (SD)) | 2.67 (0.13) | 2.67 (0.11) | 2.67 (0.13) | 2.64 (0.11) | 1.198 | 0.309 | 0.387 |
| Left temporalpole (mean (SD)) | 3.55 (0.31) | 3.54 (0.30) | 3.42 (0.59) | 3.51 (0.32) | 1.189 | 0.313 | 0.391 |
| Right precentral (mean (SD)) | 2.05 (0.19) | 2.03 (0.17) | 2.00 (0.15) | 2.01 (0.13) | 1.181 | 0.316 | 0.395 |
| Right posteriorcingulate (mean (SD)) | 2.25 (0.14) | 2.23 (0.13) | 2.22 (0.15) | 2.23 (0.11) | 1.003 | 0.391 | 0.413 |
| Right fusiform (mean (SD)) | 2.48 (0.15) | 2.46 (0.12) | 2.46 (0.12) | 2.45 (0.13) | 1.015 | 0.386 | 0.435 |
| Left lateraloccipital (mean (SD)) | 2.13 (0.12) | 2.12 (0.10) | 2.11 (0.11) | 2.11 (0.13) | 0.897 | 0.442 | 0.442 |
| Left isthmuscingulate (mean (SD)) | 2.30 (0.17) | 2.28 (0.17) | 2.24 (0.19) | 2.27 (0.18) | 1.087 | 0.354 | 0.442 |
| Right postcentral (mean (SD)) | 1.92 (0.14) | 1.91 (0.14) | 1.93 (0.14) | 1.88 (0.12) | 1.320 | 0.267 | 0.445 |
| Right inferiorparietal (mean (SD)) | 2.39 (0.13) | 2.38 (0.11) | 2.37 (0.12) | 2.36 (0.10) | 1.163 | 0.323 | 0.448 |
| Right paracentral (mean (SD)) | 2.08 (0.23) | 2.07 (0.20) | 2.01 (0.18) | 2.05 (0.17) | 1.064 | 0.364 | 0.455 |
| Right rostralmiddlefrontal (mean (SD)) | 2.30 (0.11) | 2.29 (0.10) | 2.26 (0.14) | 2.29 (0.09) | 1.016 | 0.385 | 0.481 |
| Left medialorbitofrontal (mean (SD)) | 2.39 (0.10) | 2.37 (0.11) | 2.34 (0.16) | 2.37 (0.11) | 1.543 | 0.202 | 0.505 |
| Right isthmuscingulate (mean (SD)) | 2.20 (0.17) | 2.19 (0.18) | 2.19 (0.20) | 2.16 (0.17) | 1.214 | 0.303 | 0.506 |
| Left superiortemporal (mean (SD)) | 2.64 (0.13) | 2.62 (0.12) | 2.62 (0.14) | 2.62 (0.15) | 0.973 | 0.405 | 0.506 |
| Right caudalanteriorcingulate (mean (SD)) | 2.27 (0.22) | 2.27 (0.23) | 2.20 (0.27) | 2.29 (0.21) | 0.767 | 0.513 | 0.513 |
| Left insula (mean (SD)) | 3.05 (0.18) | 3.03 (0.18) | 2.97 (0.20) | 3.00 (0.17) | 1.525 | 0.207 | 0.517 |
| Right caudalmiddlefrontal (mean (SD)) | 2.42 (0.15) | 2.41 (0.13) | 2.40 (0.13) | 2.39 (0.12) | 0.923 | 0.429 | 0.536 |
| Left supramarginal (mean (SD)) | 2.41 (0.11) | 2.40 (0.11) | 2.41 (0.15) | 2.39 (0.10) | 1.111 | 0.344 | 0.573 |
| Left fusiform (mean (SD)) | 2.48 (0.12) | 2.47 (0.11) | 2.48 (0.14) | 2.46 (0.14) | 0.720 | 0.540 | 0.590 |
| Right medialorbitofrontal (mean (SD)) | 2.44 (0.11) | 2.43 (0.11) | 2.41 (0.14) | 2.41 (0.11) | 0.828 | 0.479 | 0.599 |
| Right middletemporal (mean (SD)) | 2.73 (0.13) | 2.72 (0.12) | 2.71 (0.13) | 2.71 (0.11) | 1.002 | 0.391 | 0.612 |
| Left parsopercularis (mean (SD)) | 2.53 (0.12) | 2.52 (0.12) | 2.54 (0.13) | 2.50 (0.10) | 0.784 | 0.503 | 0.617 |
| Right rostralanteriorcingulate (mean (SD)) | 2.62 (0.16) | 2.62 (0.17) | 2.57 (0.20) | 2.59 (0.16) | 0.831 | 0.477 | 0.637 |
| Right superiorfrontal (mean (SD)) | 2.55 (0.14) | 2.54 (0.12) | 2.53 (0.16) | 2.52 (0.10) | 0.764 | 0.514 | 0.643 |
| Left caudalanteriorcingulate (mean (SD)) | 2.29 (0.24) | 2.30 (0.23) | 2.27 (0.33) | 2.26 (0.22) | 0.580 | 0.629 | 0.655 |
| Left transversetemporal (mean (SD)) | 2.13 (0.19) | 2.11 (0.19) | 2.08 (0.22) | 2.11 (0.18) | 0.717 | 0.542 | 0.677 |
| Right entorhinal (mean (SD)) | 3.57 (0.30) | 3.55 (0.31) | 3.53 (0.35) | 3.52 (0.29) | 0.488 | 0.690 | 0.690 |
| Right temporalpole (mean (SD)) | 3.59 (0.31) | 3.56 (0.31) | 3.60 (0.26) | 3.54 (0.28) | 0.644 | 0.587 | 0.691 |
| Right supramarginal (mean (SD)) | 2.41 (0.14) | 2.40 (0.12) | 2.43 (0.11) | 2.39 (0.11) | 0.671 | 0.570 | 0.712 |
| Left parahippocampal (mean (SD)) | 2.21 (0.20) | 2.19 (0.18) | 2.25 (0.24) | 2.19 (0.22) | 0.580 | 0.628 | 0.786 |
| Right bankssts (mean (SD)) | 2.54 (0.17) | 2.53 (0.14) | 2.53 (0.17) | 2.52 (0.14) | 0.577 | 0.630 | 0.788 |
| Left parsorbitalis (mean (SD)) | 2.53 (0.13) | 2.54 (0.14) | 2.57 (0.18) | 2.53 (0.14) | 0.557 | 0.643 | 0.804 |
| Left parstriangularis (mean (SD)) | 2.41 (0.12) | 2.40 (0.12) | 2.41 (0.16) | 2.39 (0.11) | 0.346 | 0.792 | 0.859 |
| Right frontalpole (mean (SD)) | 2.39 (0.24) | 2.41 (0.22) | 2.41 (0.26) | 2.39 (0.19) | 0.220 | 0.882 | 0.882 |
| Left frontalpole (mean (SD)) | 2.44 (0.27) | 2.44 (0.23) | 2.43 (0.23) | 2.46 (0.21) | 0.177 | 0.912 | 0.912 |
| Left rostralanteriorcingulate (mean (SD)) | 2.57 (0.16) | 2.56 (0.16) | 2.53 (0.20) | 2.57 (0.15) | 0.546 | 0.651 | 0.969 |
| Right parsopercularis (mean (SD)) | 2.51 (0.13) | 2.50 (0.13) | 2.51 (0.14) | 2.50 (0.12) | 0.188 | 0.905 | 0.995 |
| Right parstriangularis (mean (SD)) | 2.40 (0.12) | 2.40 (0.12) | 2.41 (0.13) | 2.40 (0.11) | 0.021 | 0.996 | 0.996 |
| Note: bold fonts suggest P_fdr_<0.05 | | | | | | | |

| Supplemental Table 4. Association of social mobility and brain cognitie outcomes in full adjusted diagonal reference models. (N= 771) | | | |
| --- | --- | --- | --- |
|  | Global cognition | Regional gray matter volume | Regional cortical thickness |
|  | Beta [95%CI] | Beta [95%CI] | Beta [95%CI] |
| Upward mobility | -.412 [-1.052, .227] | -4.827 [-13.289, 3.634] | -.054 [-.291, .183] |
| Downward mobility | .010 [-.498 .518] | -3.093 [-9.495, 3.309] | -.120 [-.054, .107] |
| Mobility distance | .128 [-.105, .362] | .276 [-2.602, 3.155] | .011 [-.003, .009] |
| Married or cohabiting | -.0805 [-.411, .250] | .612 [-3.063, 4.287] | .019 [-.082, .122] |
| Education mobility | -.028 [-.072, .015] | **.533 [.058, 1.007]*** | .012 [-.002, .025] |
| Fluid intelligence | **.046 [.027, .065]*** | **.518 [.042, .996]*** | .003 [-.002, .008] |
| Diabetes | -.077 [-.559, .403] | -2.870 [-8.182, 2.441] | .0001 [-.147, .149] |
| Stage 2 hypertension | -.167 [-.560, .2242] | 2.886 [-1.423, 7.196] | .077 [-.043, .197] |
| Depression | -.037 [-.564, .490] | -1.158 [-6.878, 4.561] | -.086 [-.246, .073] |
| Obesity | **-.475 [-.883, -.066]*** | -2.915 [-7.410, 1.580] | -.075 [-.200 .052] |
| *P<0.05, ** P<0.01. | |  |  |

| Supplemental Table 5. Linear mixed models estimation on social mobility and cognitive change during follow-up , Whitehall II Study. (N= 771) | | | |
| --- | --- | --- | --- |
|  | Unadjusted | Model 1 | Model 2 |
|  | Beta [95%CI] | Beta [95%CI] | Beta [95%CI] |
| Stable high | 0 [Reference] | 0 [Reference] | 0 [Reference] |
| Downward mobility | -0.435 [-1.213,0.343] | -0.388 [-1.181,0.404] | -0.453 [-1.251, 0.346] |
| Stable low | - 0.184 [-1.689,1.322] | -0.141 [-1.672,1.390] | **-3.526 [-5.967,-1.084]*** |
| Upward mobility | 0.055 [-0.447,0.557] | 0.091 [-0.420,0.601] | -0.033 [-0.588,0.520] |
| Stable high * time | 0 [Reference] | 0 [Reference] | 0 [Reference] |
| Downward mobility * time | 0.011 [-0.024,0.047] | 0.011[-0.025,0.046] | 0.014 [-0.023,0.050] |
| Stable low * time | 0.014 [-0.053,0.082] | 0.013 [-0.054,0.081] | 0.147 [-0.040,0.254] |
| Upward mobility * time | 0.000 [-0.031,0.015] | -0.007[-0.031,0.016] | -0.003 [-0.028,0.023] |
| Time | **-0.186 [-0.284,-0.088]**** | **-0.189 [-0.270,-0.065]**** | **-0.149 [-0.267,-0.031]**** |
| Time-squared | **0,003 [0.001,0.006]**** | **0.003 [0.001,0.005]**** | **0.003 [0.001,0.005]**** |
| Goodness of fit |  |  |  |
| AIC | 9293.4 | 9256.758 | 6095.171 |
| BIC | 9371.242 | 9358.552 | 6228.849 |
| Notes: AIC = Akaike information criterion; BIC = Bayesian information criterion;**P*< .05, ***P*< .01, ****P*< .001. | | | |
| Model 1 corrected age, gender, the total value of 48 gay matter volume and four cortical thickness regions detected related to social mobility. Model 2 corrected covariates in Model 1 and lifestyle covariates including marital status, obesity, depression, hypertension, and fluid intelligence. | | | |
